# Diet Quality of Young Adults Enrolling in TXT2BFiT, a Mobile Phone-Based Healthy Lifestyle Intervention

**DOI:** 10.2196/resprot.4484

**Published:** 2015-05-27

**Authors:** Monica Marina Nour, Kevin McGeechan, Annette TY Wong, Stephanie R Partridge, Kate Balestracci, Rajshri Roy, Lana Hebden, Margaret Allman-Farinelli

**Affiliations:** ^1^Charles Perkins CentreSchool of Molecular BioscienceUniversity of SydneySydney, NSWAustralia; ^2^School of Public HealthUniversity of SydneySydney, NSWAustralia

**Keywords:** food habits, young adults, overweight, mHealth, eHealth, telemedicine

## Abstract

**Background:**

Young adulthood is associated with poor dietary habits and vulnerability to weight gain. Population studies have revealed that inadequate fruit and vegetable intake, excessive sugar-sweetened beverages, and frequent takeaway food consumption are dietary habits requiring intervention.

**Objective:**

The aim was to examine the dietary patterns and diet quality of overweight young adults on enrollment into a mobile phone–based healthy lifestyle (mHealth) intervention, TXT2BFiT.

**Methods:**

Baseline diets were analyzed using the online Dietary Questionnaire for Epidemiological Studies version 2. The Healthy Eating Index for Australians (HEIFA) based on the 2013 Dietary Guidelines, was used to rate individual diets according to intake of core foods and deleterious nutrients including sugar, sodium, saturated fat, and alcohol. Findings were compared with the 2011 Australian National Nutrition and Physical Activity Survey (NNPAS). Gender differences were assessed with *t* tests and chi-square tests. ANOVA models were used to determine linear trends of core and noncore food intake and nutrients across quartiles of HEIFA scores. Associations between HEIFA score, sugar-sweetened beverages, and takeaway food consumption were assessed using linear regression analysis.

**Results:**

Diets of 230 participants (females: n=141; males: n=89; body mass index: mean 27.2, SD 2.5 kg/m^2^) were analyzed. The mean diet quality score was 45.4 (SD 8.8, range 21.7-77.0) out of 100 points, with no significant difference between genders. Compared with the NNPAS data for adults aged 19-30 years, this cohort had a lower intake of some core foods and higher intake of alcohol and saturated fat. Better quality diets were associated with higher intakes of fruits, vegetables, and wholegrains (*P*<.001). Takeaway food (*P*=.01) and sugar-sweetened beverage consumption (*P*<.001) were negatively associated with diet quality.

**Conclusions:**

Overweight young adults had poorer diets compared with the reference Australian population within the same age group. This study reinforces that gender-specific interventions are required, as is the current practice in TXT2BFiT, with a need to reduce sodium and alcohol intake in males and sugar intake in females. It also confirms the need to increase fruit and vegetable intake and reduce takeaway food consumption in this population, with additional focus on saturated fat and wholegrain intake.

## Introduction

It is widely recognized that poor diet quality and physical inactivity can increase the risk of becoming overweight and developing chronic diseases [1,2]. In developed countries, reduced levels of incidental activity combined with a changing food supply consisting of many energy-dense, nutrient-poor (EDNP) options, also known as discretionary or noncore foods, may have contributed to the rising prevalence of obesity [3,4]. Young adults are at the greatest risk of increasing body weight as they transition to independence, become responsible for their food choices, and are more likely to develop poor eating habits [5-7]. Data from the latest Australian Health Survey revealed that young adults have the lowest fruit and vegetable intake and obtain a greater percentage of their total energy from discretionary foods and sugar-sweetened beverages [8].

Despite the poor dietary habits and vulnerability of this population to weight gain, prevention initiatives targeting this age group are lacking [9,10]. Additionally, the number of Australian studies investigating the dietary patterns of overweight young adults is limited [11,12]. The existing research indicates that the diet quality of overweight or obese young adults varies from those within the healthy weight range [12]. As weight gain in young adulthood tends to persist throughout adulthood [13], examining the diets of this population will inform age-appropriate strategies to prevent weight gain and reduce the risk of chronic disease in later life.

Diet quality indexes are a commonly used methodological approach in exploring the dietary patterns of populations [12,14,15]. They are designed to compare dietary intakes with current healthy eating guidelines and recommendations. The outcome is a summary measure of overall diet quality that represents a collection of scores applied to intake of dietary components deemed to be in-line with the guidelines. This holistic approach to diet characterization is considered superior to alternative methods which explore individual nutrients because it is the whole diet which impacts health, not just specific foods, food groups, or nutrients [16,17].

The TXT2BFiT mobile phone–based (mHealth) intervention uses technology to provide an easily accessible and convenient lifestyle program aimed to prevent weight gain in young adults aged 18-35 years. This population provides the opportunity to study food intakes of overweight young adults seeking assistance to change.

The primary objective of this study was to classify the quality of the baseline diets of TXT2BFiT participants using a modified version of the Healthy Eating Index for Australians (HEIFA) [18]. The secondary objective was to compare the dietary patterns (core, noncore food, and micro- and macronutrient intake) of overweight young adults to the nationally representative sample of adults aged 19-30 years who participated in the National Nutrition and Physical Activity Survey (NNPAS). Finally, this study sought to explore the amount of sugar-sweetened beverages and the frequency of takeaway food consumption among this cohort, while examining how this varies with diet quality.

## Methods

### Participants

Materials and methods of the TXT2BFiT Randomized Controlled Trial were approved by the University of Sydney Human Research Ethics Committee (approval number 13698). Baseline dietary data were collected from 250 young adults enrolled in the TXT2BFiT healthy lifestyle program. TXT2BFiT is tailored to address the health-related beliefs, barriers, and sociocultural norms of young adults aged between 18 and 35 years. In combination with telephone counseling and website and mobile phone app use, participants received text messages targeting behaviors such as inadequate fruit and vegetable intake, physical inactivity, and excessive sugar-sweetened beverages, alcohol, and energy-dense takeaway food consumption. Participants were recruited from the greater Sydney area in New South Wales, Australia [19,20]. They completed a baseline survey and a food frequency questionnaire (FFQ). The baseline survey included questions on frequency of sugar-sweetened beverages, water, and takeaway food consumption. Details of the specific information collected are published elsewhere [19]. Participants who did not complete the baseline survey (n=2) or made “serious” errors in their responses on the FFQ (n=3) were excluded, as well as those determined as over- and underreporters (n=15), defined as energy intake basal metabolic rate (BMR) of <0.5 or >2.0 (BMR was calculated using the Schofield equation based on body weight, age, and gender) [21,22].

### Dietary Assessment

Baseline self-reported dietary intake was measured online using a FFQ known as the Dietary Questionnaire for Epidemiological Studies version 2 (DQESv2) created by the Cancer Council Victoria [19,23]. This 74-item questionnaire was used to gather the respondent’s usual consumption of food and alcohol in the last month. Although this tool was originally designed to measure intake over the preceding 12 months, it was previously validated against 5-day weighed food records to measure intake over 1 month for Australian young adults. It was found that the DQESv2 is a valid measure of all nutrients studied at the group level and has good reproducibility [24,25]. Because the DQESv2 does not measure sugar-sweetened beverage intake, a single-item question that asked respondents “On average how much sugary drinks do you usually drink per week” was used to determine consumption of sugar-sweetened beverages. Respondents were asked to consider intake of soft drinks, energy drinks, sport drinks, cordials, vitamin waters, and iced teas, but not diet, low joule, or artificially sweetened drinks for which a separate response category was provided. The reproducibility and reliability of this question was tested previously against weighed food records using interclass correlations and weighted kappa statistic. Analyses revealed that the short question is a valid tool for classing intake into categories at the population level and has good reproducibility allowing for variation in sugar-sweetened beverages overtime to be detected (unpublished results).

### Diet Quality Scoring Using the HEIFA

A modified version of the HEIFA (Multimedia Appendix 1) based on adherence to the most recent Dietary Guidelines for Australian Adults 2013 (DGAA) and The Australian Guide to Healthy Eating 2013 (AGHE) [26], was used to determine the diet quality of participants based on data from both the online DQESv2 and the baseline questionnaire. Because there is currently no agreement on how EDNP foods should be defined [27,28], the AGHE was used to classify foods as either core or noncore [26].

The modified HEIFA presented in Multimedia Appendix 1, assessed 11 components: the 5 food groups, consumption of discretionary (noncore) foods, and intake of water, alcohol, fats, total sugars, and sodium. The maximum score was awarded to participants who met the specified criteria based on the guidelines. Prorated scores were given to intakes below the recommendation. Points were awarded for choosing a variety of fruit and vegetables and for consuming low-fat dairy. The total score ranged from zero to 100, with higher scores reflecting better adherence to the dietary guidelines.

### Statistical Analysis

All statistical analyses were conducted using SPSS version 22 for Windows (IBM Corp, Armonk, NY, USA). The normality of the distribution of diet quality scores was assessed before analyses. As the data was found to be normally distributed, no transformations were required. The number of participants who attained the maximum score on each dietary component assessed by the HEIFA was tallied to determine the percentage of participants meeting each guideline. Quartiles of HEIFA scores were then created for the overall cohort, in which quartile 1 (Q1) indicated a diet least consistent and quartile 4 (Q4) represented a diet most consistent with the DGAA. The mean diet quality score and individual dietary component score was determined for the cohort and across quartiles. A *t* test and chi-square test for proportions was used to examine differences between genders. A polynomial 1-way analysis of variance (ANOVA) was used to identify linear trends in intakes of core foods, noncore foods, and selected nutrients across quartiles. Macronutrient and core and noncore food intakes were calculated as the mean percent of total energy intake for both genders. The calorie content of core and noncore foods selected were calculated as the mean of all brands presented in the Australian Food and Nutrient Database (AUSNUT 2011-2013) [29]. Intake of core foods, noncore foods, and nutrients were compared to corresponding data from the representative sample of adults age 19-30 years who participated in the NNPAS [8,30]. Finally, linear regression analyses were employed to explore the associations between diet quality, sugar-sweetened beverage intake, and frequency of takeaway food consumption.

## Results

### Participants

The data from 141 females and 89 males (N=230) were included in the analyses. These participants were aged between 18-34 years, with a mean age of 27.7 years (SD 4.9). The mean body mass index (BMI) was 27 kg/m^2^ (SD 2.5) with 20.4% (47/230) of participants classed as obese and 63.5% (146/230) as overweight. The mean total energy intake per day was 2123 kcal (SD 865) and 1706 kcal (SD 630) for men and women, respectively. This was lower than that of the mean total energy intakes of the NNPAS population (2632 kcal/day for males and 1881 kcal/day for females).

### Diet Quality

#### Overview

In this population of young adults, the mean diet quality score was 45.4 (SD 8.8, range 21.7-77.0), with a slightly higher average score in males than females (males: mean 46.6, SD 9.2, range 26.8-77.0; females: mean 44.6, SD 8.5, range 21.7-65.9). There was minimal variation in the individual dietary components of HEIFA between genders, with the exception of sodium, sugar, and alcohol intake (Table 1). As seen in Table 1, a greater proportion of women met the dietary guidelines for sodium (*P*<.05) and alcohol (*P*<.01), whereas a greater proportion of males met the guidelines for sugar (*P*<.01). Overall, there were no dietary guidelines that were well met by the study population. The highest individual component score attained was for meats and meat alternatives. The percentage of the sample scoring zero in a category was highest for saturated fat (76.1%, 175/230) and vegetable variety (94.3%, 217/230).

**Table 1:** The number (%) of participants meeting each dietary guideline.

Dietary component	Cohort (n=230)		Males (n=89)		Females (n=141)		*P*
	Score, mean (SD)	Met guideline, n (%)	Score, mean (SD)	Met guideline, n (%)	Score, mean (SD)	Met guideline, n (%)	
Discretionary foods^a^	4.3 (3.1)	0 (0.0)	4.6 (3.1)	0 (0)	4.0 (3.1)	0 (0.0)	>.99
Vegetables^a^	1.0 (1.1)	0 (0.0)	1.0 (1.1)	0 (0)	1.1 (1.1)	0 (0.0)	.30
Fruit^a^	5.2 (3.6)	73 (31.7)	4.9 (3.6)	26 (29)	5.3 (3.7)	47 (33.3)	.59
Breads & cereals^a^	4.8 (2.8)	14 (6.5)	5.1(2.8)	8 (9)	4.7 (2.9)	7 (5.0)	.20
Meat & alternatives^a^	6.7 (3.3)	87 (37.8)	6.7 (3.5)	39 (44)	6.7 (3.1)	48 (34.0)	>.99
Dairy & alternatives^a^	5.6 (3.0)	15 (6.5)	5.3 (3.0)	5 (6)	5.8 (2.9)	10 (7.1)	.17
Water^b^	2.1 (1.3)	13 (5.6)	2.2 (1.3)	7 (8)	2.0 (1.3)	6 (4.3)	.42
Fat^a^	1.8 (2.2)	2 (0.9)	1.5 (2.1)	0 (0)	2.1 (2.3)	2 (1.4)	.15
Sodium^a^	3.9 (4.0)	51 (22.2)	2.8 (3.5)	11 (12)	4.6 (4.0)	40 (28.4)	<.05
Total sugars^a^	5.1 (4.0)	75 (32.6)	6.6 (3.7)	42 (47)	4.2 (4.0)	33 (23.4)	<.05
Alcohol^b^	3.8 (2.1)	176 (76.5)	3.4 (2.4)	60 (67)	4.1 (1.9)	116 (82.3)	<.01

^a^ Scored out of 10.

^b^ Scored out of 5.

#### Intake of Core and Noncore Foods

Tables 2 and 3 compare the total mean intakes of core and noncore foods of TXT2BFiT participants to that of Australians aged 19-30 years who participated in the NNPAS. Mean baseline vegetable intake in our study population was similar between genders; however, males consumed more fruit. Compared to NNPAS participants, this cohort consumed fewer vegetables and more fruit. The dietary guidelines recommend that mostly wholegrain cereals are consumed. This cohort’s overall wholegrain intake was poor, with 33.5% (77/230) of the cohort consuming mostly (>50%) wholegrain bread and cereal products. Dairy intake was higher in this cohort with 59.1% (136/230) consuming low-fat (skim or reduced fat) milk. Overall, the current study population obtained 10% more energy from core foods compared to Australians aged 19-30 years who took part in the NNPAS. Although the TXT2BFiT participants consumed less fruit juice and sugar-sweetened beverages than the NNPAS population, they had a higher intake of alcohol. Frequency of takeaway food consumption was high with 45.7% (105/230) and 13.0% (30/230) of the cohort consuming takeaway foods 2-3 times and 4-5 times per week, respectively.

**Table 2:** Mean intake (grams) of selected core foods for males and females and their mean percentage contribution to total energy intake using results from the Dietary Questionnaire for Epidemiological Studies version 2 (DQESv2), compared to the averages of Australians aged 19-30 years who took part in the National Nutrition and Physical Activity Survey (NNPAS).

Core food groups		Total		Male		Female	
		DQESv2	NNPAS^a^	DQESv2	NNPAS^a^	DQESv2	NNPAS^a^
**Intake (g), mean (SD)**							
	Vegetables	116.8 (78.9)	172.8	119.8 (93.1)	172.9	115.0 (68.8)	173.0
	Fruit^b^	209.1 (153.7)	109.5	228.3 (168.3)	106.3	197.0 (143.1)	112.9
	Breads and cereals	242.5 (148.0)	171.5	272.6 (158.6)	209.3	223.5 (138.1)	132.1
	Wholegrain cereals^c^	90.1 (86.0)	—	87.9 (97.4)	—	91.5 (78.2)	—
	Meat and alternatives	190.1 (116.1)	221.5	233.7 (146.1)	278.2	162.5 (81.7)	162.6
	Dairy and alternatives	337.3 (168.8)	242.1	335.8 (170.5)	280.7	338.2 (168.3)	201.9
	Low-fat dairy^d^	172.4 (172.2)	—	164.9 (180.5)	—	177.1 (167.2)	—
**% Energy, mean (SD)**							
	Vegetables^e^	3.6 (2.9)	7.5	3.4 (3.6)	6.8	3.7 (2.3)	8.7
	Fruit^b^	5.9 (5.0)	3.1	5.6 (4.9)	2.7	6.1 (5.1)	3.6
	Breads and cereals	24.4 (11.5)	16.5	24.9 (12.6)	17.0	24.1 (10.7)	15.7
	Meat and alternatives^f^	21.8 (8.6)	19.4	23.3 (9.5)	20.6	20.9 (7.9)	17.2
	Dairy and alternatives^g^	10.5 (7.1)	9.9	9.4 (6.9)	9.9	11.2 (7.2)	9.9
	TOTAL	66.2 (15.5)	56.4	66.6 (16.1)	57.0	66.0 (15.1)	55.1

^a^ National Nutrition and Physical Activity Survey (NNPAS) results for participants aged 19-30 years. Mean intake (g) obtained from data cube table 5 and mean % energy from data cube table 8 [8]. SDs not available for NNPAS data.

^b^ Excluding fruit juice.

^c^ Includes whole meal, rye, and multigrain bread, All Bran, Bran Flakes, Weet-Bix, porridge, and muesli. Wholegrain intake not available for NNPAS population.

^d^ Low-fat dairy intake not available for NNPAS population.

^e^ Including legumes.

^f^ Including meat, poultry, game products and dishes, egg products and dishes, fish, seafood product and dishes, legumes and nuts.

^g^ Including milk products and dishes and milk substitutes.

**Table 3:** Mean intake (grams) of selected noncore foods for males and females and their mean percentage contribution to total energy intake using results from the Dietary Questionnaire for Epidemiological studies version 2 (DQESv2), compared to the averages of Australians aged 19-30 years who took part in the National Nutrition and Physical Activity Survey (NNPAS).

Noncore foods		Total		Male		Female	
		DQESv2	NNPAS^a^	DQESv2	NNPAS^a^	DQESv2	NNPAS^a^
**Intake (g), mean (SD)**							
	Crackers	7.4 (12.5)	3.5	6.9 (13.9)	9.2	7.7 (11.7)	3.7
	Sweet biscuits	8.7 (14.2)	7.8	7.5 (12.5)	3.2	9.4 (15.3)	6.3
	Cake	16.2 (20.7)	17.2	15.8 (23.5)	15.3	16.4 (18.7)	19.3
	Chocolate	17.6 (22.9)	6.8	13.8 (19.5)	4.6	20.1 (23.4)	9.0
	Crisps	5.3 (7.6)	3.8	4.9 (6.5)	5.4	5.6 (8.3)	2.1
	Jam	3.5 (6.2)	1.6	2.8 (4.5)	2.2	3.9 (6.9)	1.0
	Margarine	1.3 (5.5)	1.9	1.8 (5.7)	1.8	0.9 (4.9)	2.0
	Butter	3.4 (7.2)	1.6	4.1 (8.4)	1.6	3.0 (6.2)	1.6
	Ice cream	11.6 (23.0)	—^c^	9.3 (10.8)	—	13.1 (28.3)	—
	Sausage	8.2 (11.7)	10.2	10.7 (13.7)	13.4	6.6 (10.1)	6.8
	Salami	3.5 (8.25)	—	4.2 (9.9)	—	3.1 (7.1)	—
	Meat pie	14.0 (16.4)	—	14.8 (15.4)	—	13.6 (17.1)	—
	Pizza	28.9 (33.9)	—	41.2 (43.7)	—	21.1 (23.2)	—
	Hamburger	15.0 (20.7)	—	22.8 (25.3)	—	10.1 (15.4)	—
	Hot chips	13.1 (12.5)	—	16.0 (14.4)	—	11.3 (10.8)	—
	Fruit juice (mL)	113.0 (175.7)	126.8	124.7 (192.3)	149.7	66.2 (82.6)	102.8
	Alcohol	13.9 (19.7)	10.8	18.9 (22.9)	14.4	10.7 (1.4)	7.0
	Sugar-sweetened beverages (mL) ^b^	81.5 (101.3)	309.4	71.4 (109.1)	389.9	67.9 (93.8)	225.4
**% Energy, mean (SD)**							
	Crackers	1.5 (1.9)	0.4	1.2 (1.9)	0.4	1.7 (1.8)	0.5
	Sweet biscuits	2.0 (2.6)	1.6	1.6 (2.3)	1.7	2.2 (2.7)	1,6
	Cake	2.8 (3.0)	2.6	2.4 (3.0)	2.1	3.1 (2.9)	3.5
	Chocolate	4.6 (5.3)	1.5	3.1 (3.7)	0.8	5.6 (6.2)	2.4
	Crisps	1.4 (2.0)	0.9	1.2 (1.3)	1.0	1.6 (2.3)	0.6
	Jam	0.4 (0.6)	0.2	0.3 (0.5)	0.3	0.4 (0.6)	0.2
	Margarine	0.3 (1.5)	0.5	0.4 (1.4)	0.4	0.3 (1.4)	0.6
	Butter	1.2 (2.5)	0.5	1.3 (2.5)	0.4	1.2 (2.5)	0.6
	Ice cream	1.2 (1.4)	—^c^	0.9 (1.0)	—	1.4 (2.0)	—
	Sausage	0.9 (1.2)	1.1	1.1 (1.3)	1.3	0.8 (1.0)	0.8
	Salami	0.7 (1.4)	—	0.7 (1.2)	—	0.7 (1.5)	—
	Meat pie	1.7 (2.0)	—	1.5 (1.4)	—	1.8 (2.3)	—
	Pizza	3.7 (3.3)	—	4.6 (3.6)	—	3.1 (2.9)	—
	Hamburger	1.8 (2.2)	—	2.5 (2.6)	—	1.4 (1.8)	—
	Hot chips	1.7 (1.5)	2.5	1.7 (1.4)	2.7	1.6 (1.6)	2.3
	Fruit juice	0.2 (1.3)	1.1	0.2 (1.0)	1.2	0.2 (1.4)	1.1
	Alcohol	5.1 (7.0)	2.8	6.0 (7.1)	3.5	4.7 (6.9)	2.1
	Sugar-sweetened beverages	1.5 (2.5)	3.9	1.6 (2.6)	4.5	1.4 (2.4)	3.2
	TOTAL	33.2 (12.4)	35.9	33.0 (12.5)	36.4	33.5 (12.7)	35.2

^a^ National Nutrition and Physical Activity Survey (NNPAS) results for participants aged 19-30 years. Mean intake (g) obtained from data cube table 5 and mean % energy from data cube table 9 [8]. SD’s not available.

^b^ Excluding artificially sweetened drinks. Includes soft drink, cordials, flavored mineral water, electrolyte, energy, and fortified drinks. Measured using a single-item question not the DQESv2.

^c^ Ice cream data from the NNPAS has not been included because the category of frozen milk products also measures frozen yogurt intake, limiting comparability.

#### Change Across Quartiles

As displayed in Table 4, the mean intake of core food groups increased as diet quality improved with the exception of dairy. Higher intake of low-fat dairy was significantly associated with higher HEIFA scores. Additionally, noncore food and sugar-sweetened beverage consumption was lowest for participants with the highest diet quality, indicating an association between lower intakes of these less desirable items and higher quality diets. Higher diet quality scores were also associated with higher intake of iron, vitamin C, zinc, and folate. Finally, negative associations between HEIFA score, takeaway food consumption (*P*=.01), and sugar-sweetened beverage intake (*P*<.001) were found (not displayed in table).

**Table 4:** Mean diet quality (Healthy Eating Index for Australians, HEIFA) scores across quartiles and the associated mean intake of core and noncore foods.

Dietary component		Quartile				*P* ^a^
		1	2	3	4	
HEIFA score, mean (SD)		34.0 (4.1)	42.4 (1.6)	48.1 (2.0)	56.4 (4.9)	
Energy (kcal/day), mean (SD)		1865 (852)	1886 (660)	1747 (621)	1970 (868)	
**Core foods, mean (SD)**						
	Vegetables (g)	77.3 (55.9)	107.9 (68.2)	125.4 (65.7)	154.7 (99,4)	<.001
	Fruit (g)	126.2 (87.0)	176.8 (118.4)	240.6 (174.2)	289.0 (167.2)	<.001
	Breads and cereals (g)	182.8 (88.0)	211.5 (127.1)	278.0 (159.3)	295.3 (173.9)	<.001
	Wholegrains^b^ (g)	61.6 (56.7)	72.7 (62.9)	96.2 (83.6)	128.8 (114.0)	<.001
	Meat and alternatives (g)	149.5 (85.9)	176.6 (96.7)	216.8 (154.8)	215.5 (102.7)	<.001
	Dairy and alternatives (g)	322.7 (181.2)	336.6 (195.5)	336.1 (142.9)	353.0 (153.5)	.42
	Low-fat dairy (g)	125.7 (181.3)	171.0 (173.2)	170.6 (159.7)	219.8 (166.0)	.006
**Noncore foods, mean (SD)**						
	Discretionary foods^c^ (g)	64.6 (55.2)	70.8 (59.3)	65.0 (69.7)	58.2 (36.2)	.004
	Sugar-sweetened beverages (mL)	109.7 (118.2)	84.7 (107.7)	81.1 (94.5)	51.4 (73.9)	<.001
**Nutrients, mean (SD)**						
	Iron (mg)	10.4 (4.0)	11.9 (5.5)	12.5 (5.0)	13.3 (4.6)	<.001
	Vitamin C (mg)	60.4 (28.3)	77.9 (32.1)	104.7 (74.1)	105.7 (49.8)	<.001
	Zinc (mg)	10.2 (4.2)	11.2 (4.9)	12.7 (6.5)	12.4 (4.5)	.008
	Folate (μg)	185.5 (66.5)	223.0 (92.5)	240.9 (97.5)	263.9 (91.7)	.02
	Calcium (mg)	818.2 (348.3)	856.6 (363.3)	891.0 (318.3)	890.1 (312.1)	.31

^a^ *P* for linear trend.

^b^ Includes whole meal, rye and multigrain bread, All Bran, Bran Flakes, Weet-Bix, porridge, and muesli.

^c^ Includes all noncore foods measured by the DQESv2.

#### Macronutrient Distribution

As displayed in Table 5, the mean percentage intake from protein was within the acceptable macronutrient distribution range (AMDR). The mean percentage intake from total fat fell on the upper limit of the AMDR (mean 35%), with a high percentage derived from saturated fats. The cohort’s intake of carbohydrates (40%) was below the AMDR (45%-65%). Despite this, the highest percentage of energy in the diet was derived from carbohydrates (40%), with a high percentage from sugars (17.5%). There were no large variations in macronutrient intake between males and females; however, males exceeded the maximum recommended percentage energy from alcohol. The cohort’s intake of fiber was lower than the NNPAS population, with females meeting 65% and males meeting 54% of the recommended daily intake.

**Table 5:** The mean % energy intake from carbohydrates, fat, protein, and alcohol, and the mean (SD) fiber intake (g) of the cohort using data from the Dietary Questionnaire for Epidemiological studies version 2 (DQESv2) compared to National Nutrition and Physical Activity Survey (NNPAS) data with corresponding recommendations.

Dietary component		Recommendation	Total, mean (SD)		Male, mean (SD)		Female, mean (SD)		
			DQESv2 (N=230)	NNPAS^a^ (N=1592)	DQESv2 (n=89)	NNPAS^a^ (n=739)	DQESv2 (n=141)		NNPAS^a^ (n=853)
**% Energy**									
	From carbohydrates	45%-65%^b^	40.5 (7.4)	45.3	39.3 (8.7)	45.0	41.2 (6.4)	45.5	
	From total sugars	<15%	17.6 (5.4)	19.8	15.5 (5.3)	19.1	18.8 (5.1)	20.5	
	From starch	N/A^c^	22.7 (6.1)	24.7	23.7 (6.5)	24.9	22.1 (5.8)	24.5	
	From protein	15%-25%^b^	19.8 (3.3)	18.1	20.3 (3.6)	18.5	19.5 (3.1)	17.7	
	From fat	20%-35%^b^	35.2 (5.5)	31.2	35.0 (5.6)	30.5	35.4 (5.4)	31.9	
	From saturated fat	<10%	14.3 (3.2)	11.7	14.0 (3.3)	11.4	14.5 (3.2)	12.0	
	From monounsaturated fat	N/A	12.8 (2.3)	12.0	12.9 (2.5)	11.9	12.7 (2.2)	12.0	
	From polyunsaturated fat	7%	4.9 (1.5)	4.9	4.8 (1.5)	4.7	4.9 (1.6)	5.1	
	From alcohol	<5%	5.1 (7.0)	2.8	5.9 (7.1)	3.5	4.6 (6.9)	2.1	
**Intake (g)**									
	Fiber	28-38 g/day^d^	19.2 (8.5)	23.7	20.6 (10.1)	24.8	18.3 (7.2)		

^a^ NNPAS results for participants aged 19-30 years. Mean percentage energy for macronutrients obtained from data cube table 2 [8]. SDs not available for NNPAS data.

^b^ Acceptable macronutrient distribution range (AMDR) [31].

^c^ N/A, not available.

^d^ Lower range for females, upper range for males.

## Discussion

This study shows that diet quality was generally poor among overweight young adults with an average score half of the maximum achievable. The participant’s diets were worse than the Australian population sample of adults aged 19-30 years with lower intake of vegetables and higher intake of alcohol, noncore, and takeaway foods. This finding is consistent with previous studies showing poor diet quality in individuals with a higher BMI [13].

Although diet quality scores were very low among the study participants, this is a common trend observed when scoring diets against national guidelines [12,14,15]. Participants with diets higher in core foods and that incorporated more fruits, vegetables, and wholegrain cereals were of better quality and more nutrient-dense. This finding is consistent with prior research that found higher diet quality scores are associated with higher intakes of fruits, vegetables, iron, folate, and vitamin C in men and women [15].

Contrary to prior research [14,32], overall diet quality varied little among these overweight men and women. Further diet analyses based on core and noncore food intake did, however, reveal some differences. Males consumed greater amounts of meats and meat alternatives and females obtained a greater proportion of their total energy from dairy foods. Males also reported a higher intake of savory takeaway foods, such as pizza, hamburgers, and hot chips, whereas females favored sweet treats such as chocolate, ice cream, and sweet biscuits. Considering that savory and meat-based dishes are typically higher in salt, whereas dairy products and desserts contain more sugar, it is evident why fewer men met the guidelines for sodium and fewer women met the guidelines for sugar.

This group of young adults consumed less than a quarter of the amount of sugar-sweetened beverages reported by NNPAS participants aged 19-30 years. A high proportion of participants reported to consume no sugar-sweetened beverages or artificially sweetened versions only (23.5%, 54/230 and 17.4%, 40/230, respectively), which likely contributed to the lower mean group intake. Although sugar-sweetened beverage consumption was lower than expected, alcohol intake was high with a significantly greater proportion of men exceeding the dietary guidelines for alcohol. The link between weight management and alcohol consumption is not clearly defined; however, research has demonstrated a positive correlation between alcohol intake and obesity irrespective of the type of alcohol consumed [33,34]. These results emphasize the importance of targeting a reduction in alcohol intake as part of healthy lifestyle intervention, especially for young males who are consuming amounts substantially higher than the recommendations.

Also of concern in this group is the high intake of saturated fat. Although low-fat dairy was consumed by 59.1% of participants, butter was favored over margarine and intake is twice that of the NNPAS population. It appears these young adults consume less discretionary foods than the NNPAS representative survey group, but it is important to note that there are limited discretionary foods included in the DQESv2 and intake may be greater than estimated. It is also possible that aspects of social desirability resulted in underreporting of discretionary foods that are perceived as unhealthy. Evidence of selective underreporting has been found in both genders [35,36] and especially among obese women [37]. Although the 24-hour recall used in the NNPAS is also subject to this bias, many more of the TXT2BFiT participants were overweight, increasing the likelihood of underreporting. Despite possible underestimation, the percentage energy from noncore foods was still substantially higher than the maximum recommended limit of 20%. These results highlight that interventions targeted at increasing core foods and limiting EDNP food consumption are essential for both healthy and overweight young adults.

This study suggests that those who consume takeaway foods more frequently have lower quality diets. These results are consistent with previous research, which indicates diet quality is negatively associated with takeaway food consumption [38]. A possible cause of the negative correlation between diet quality and commercially prepared meals is the lower nutrient content of the food. Takeaway foods usually contain more total and saturated fat, and less fiber than homemade meals [39]. In this group of young adults, frequency of takeaway food consumption was high with 46% (106/230) consuming commercially prepared meals 2-3 times per week and a further 13% (30/230) consuming them 4-5 times per week. Based on these results, it is evident that the component of the TXT2BFiT healthy lifestyle intervention that aims to reduce takeaway food consumption is appropriate.

Despite the cohort consuming twice as much fruit as the NNPAS population, their diets were lower in fiber. This can be attributed to an inadequate intake of wholegrain cereals and vegetables; mostly refined breads and cereals were consumed by this cohort. Vegetable intake was also poor with approximately 1.5 servings of vegetables consumed per day, less than the 2.3 servings consumed by the NNPAS participants. However, it should be acknowledged that validation of the DQESv2 with weighed food records in young adults found fruit was overestimated in males and vegetables were underestimated in both genders [24]. This raises some uncertainty as to whether vegetable intake may be better than indicated.

Overall, the study cohort appeared to consume diets lower in energy than the NNPAS population, suggesting they may be practicing calorie restriction. The TXT2BFiT intervention is designed to improve lifestyle patterns and differs from other electronic and mobile weight management interventions that typically use calorie monitoring to instigate behavior change. This is a significant strength of the intervention because it is evident that this cohort would benefit more from strategies which encourage healthier eating habits and improved diet quality.

A number of limitations must be taken into consideration when interpreting the data. Firstly, although comparisons were made with results of NNPAS, the different dietary assessment methods, food coding, and classification procedures restrict comparability. Furthermore, the DQESv2 dietary assessment tool is limited in the number of EDNP items, only includes certain brands of cereals, fails to distinguish between lean and fatty meats, and does not measure salt added at the table or in cooking. This may have resulted in underestimation of sodium and fiber. Among the strengths of this study is that it utilizes a comprehensive diet quality index (HEIFA) which focuses on food indicators based on the most recent DGAA.

This study identifies some of the dietary improvements necessary in this population and will help focus future nutrition interventions to generate change where it is most required. The results reveal that this cohort needs support to change their dietary behaviors to limit alcohol intake and replace refined cereals with wholegrain foods and high-fiber cereals. Although the TXT2BFiT program includes a beverage app that counts alcoholic, energy, and sugary drink intake and indicates when targets are exceeded, whether this is effective in reducing the excessive intake found in this group is yet to be demonstrated.

The findings of this preliminary study will also allow monitoring of a young adult population enrolling in an mHealth program. Intervention outcomes will allow us to assess whether dietary patterns at baseline influence weight loss results postintervention. Previous studies have shown that individuals with higher diet quality scores lose more weight postintervention than those with lower scores [11].

In conclusion, the findings confirm that in the young adult population, there is a need to increase fruit and vegetable consumption and decrease energy-dense takeaway food intake. Further attention to wholegrain cereal and saturated fat intake is also indicated. Additionally, this study reinforces that gender-specific interventions are required, as is the current practice in TXT2BFiT, with a need to reduce sodium and alcohol intake in males and sugar intake in females.

## Multimedia Appendix 1

The Healthy Eating Index for Australians (HEIFA) based on the new Dietary Guidelines for Australian Adults (DGAA) (2013) using an eleven-component system of 5 food groups, 4 nutrients and a measure of variety of food intake.

## Abbreviations

AMDR: acceptable macronutrient distribution range
BMI: body mass index
BMR: basal metabolic rate
DGAA: Dietary Guidelines for Australian Adults
DQESv2: Dietary Questionnaire for Epidemiological Studies version 2
FFQ: food frequency questionnaire
HEIFA: Healthy Eating Index for Australians
NNPAS: National Nutrition and Physical Activity Survey
