# Supplementary material for: Diet Quality of Young Adults Enrolling in TXT2BFiT, a Mobile Phone-Based Healthy Lifestyle Intervention
Source: JMIR Res Protoc. 2015 May 27;4(2):e60. doi: 10.2196/resprot.4484 (PMC4463996; doi:10.2196/resprot.4484)
Supplement: Supplementary file 1 [file resprot_v4i2e60_app1.pdf]

Dietary Guidelines for Australian Adults	Indicator and Description	Composite Sub Scores[a]
To achieve and maintain a healthy weight, be physically active and choose amounts of nutritious foods and drinks to meet energy needs	**Measure of unhealthy choices, Discretionary foods:** frequency of consumption of discretionary foods per day.	**Discretionary Foods/day**:<br>**Male:**<br>0 serves/d = 10,<br>0.1-2.9 = 7.5<br>3 = 5<br>3.1-5.9 =2.5<br>≥6 serves = 0<br>**Female:**<br>0 serves = 10,<br>0.1-2.4 =7.5;<br>2.5=5;<br>2.6-4.9 =2.5<br>≥5 serves = 0
Eat plenty of vegetables of different types and colors and legumes/beans	**Total Vegetables:** servings of vegetables and legumes per day. **Variety** of vegetable servings/day	**Total vegetable serves per day**<br>**Male**:<br>≥6 serves = 5,<br>≥4.8 < 6 serves = 4,<br>≥3.6 < 4.8 serves = 3,<br>≥2.4 < 3.6 = 2,<br>≥1.0 < 2.4 serves = 1,<br>No vegetables = 0<br>**Female:**<br>≥ 5 serves = 5,<br>≥4 <5 serves = 4,<br>≥3 < 4 serves = 3<br>≥2 < 3 serves = 2,<br>≥1 < 2 serves = 1,<br>No vegetables = 0<br>**Vegetable variety score/day:**<br>≥1 serve green = 1,<br>≥1 serve orange = 1,<br>≥1 serve of cruciferous = 1,<br>≥1 serve of tuber or bulb = 1,<br>≥0·5 serves of legumes = 1
Enjoy a wide variety of fruits	**Fruit** servings of fruit per day;<br>**Variety** of fruit servings/day	**Total Fruit serves per day**:<br>≥2 serves = 5,<br>≥1.5 < 2 serves = 3.75,<br>≥1 < 1.5 serves = 2.5,<br>≥0.5 < 1 serves = 1.25,<br>≥0 < 0.5 serves = 0<br>**Fruit variety score/day**<br>2 OR MORE varieties 5 points
Enjoy a wide variety of grain (cereal) foods, mostly wholegrain and/or high cereal fibre varieties such as breads, cereals, rice, pasta, noodles, polenta, couscous, oats, quinoa and barley	**Whole grains Refined grains Cereals:** frequency of consumption of breads and cereals per day.<br>**Whole grain cereals:** proportion of wholemeal/whole grain bread consumed relative to total bread.	**Total cereals serves/d**: 6 serves = 5,<br>5 serves = 4.17,<br>4 serves = 3.34,<br>3 serves = 2.5,<br>2 serves = 1.67,<br>1 serve = 0.84<br>**Whole-grain cereal serves/d:**<br>≥ 3 serves = 5,<br>≥2.5<3 serves = 4,<br>≥2<2.5 serves = 3,<br>≥1.5<2 serves = 2,<br>1≤1.5 serve = 1,<br>No wholegrain = 0

Enjoy a wide variety of lean meats and poultry, fish, eggs, tofu, nuts and seeds, and legumes/beans	**Total protein foods, Seafood and plant proteins, Lean meat and meat alternatives:** frequency of consumption of lean meats and alternatives per day.	**Meat/Meat Alternative serves/day**<br>**Male:**<br>≥ 3 serves = 10,<br>≥ 2.5 serves = 8,<br>≥ 2 serves = 6,<br>≥1.5 serves = 4,<br>≥ 1 serves = 2,<br>≤ 0.5 serves = 0<br>**Female**:<br>≥ 2.5 serves = 10,<br>≥ 2 serves = 8,<br>≥ 1.5 serves = 6,<br>≥1 serves = 4,<br>≥ 0.5 serves = 2,<br>No Meat/Meat Alternative = 0
Enjoy a wide variety of milk, yoghurt, cheese and/or their alternatives mostly reduced fat	Dairy consumption Dairy foods/alternatives: frequency of consumption of dairy products per day	**Dairy/Dairy alternatives/day:**<br>≥ 2.5 serves = 5,<br>≥ 2 serves = 4,<br>≥ 1.5 serves = 3,<br>≥1 serves = 2,<br>≥ 0.5 serves = 1,<br>No Dairy/Dairy Alternative = 0
	Low-fat/reduced-fat dairy: type of milk usually consumed.	**Low fat dairy score**<br>Mainly skim or low fat milk consumed = 5<br>Soy milk consumed =2.5<br>Full fat milk consumed =0
Drink plenty of water[b]	Fluids: Number of cups of water consumed per day<br><br>NOTE only scored out of 5	**Cups of water/day**<br>>9 cups/day = 5,<br>8-9 cups/day = 4,<br>6-7 cups/day = 3,<br>4-5 cups/day = 2,<br>2-3 cups/day = 1<br>0-1 =0,
Limit intake of foods high in saturated fat and replace high fat foods with foods which contain predominately PUFA and MUFA	Consumption of high saturated of fat, nutrient low-density foods and fatty acids.	**Saturated fat** ≤ 10% of energy = 5, >10% to 12% =2.5 of energy >12% total energy= 0,<br>**PUFA and MUFA**<br>**Males**<br>4 serves =5<br>3 to <4 serves =3.75<br>2 to <3 serves =2.5;<br>1 to <2serve = 1.25;<br>0 to <1serves =0<br>**Females**<br>2 serves = 5<br>1.5 to <2 =3.75<br>1 to <1.5 serves =2.5;<br>0.5 to < 1serve = 1.25;<br>0 to <0.5 serves =0
Choose lower sodium options and do not add salt to foods in cooking or at the table	Sodium: total sodium intake excluding salt added in cooking and at table	**Sodium/day:**<br>0-≤70mmol (920-1610mg) = 10.,<br>70-≤100mmol (1610-2300mg) = 5<br>≥100mmol (2300mg) of Na/day = 0
Limit intake of foods containing added sugars[c]	Percentage of energy from Total sugar	<15% total energy = 10<br>>15–<20% total energy = 5<br>>20% energy = 0

If you choose to drink alcohol, limit intake	Alcohol: frequency of consumption of all alcoholic beverages per day.	≤ 2 per day = 5<br>>2 per day = 0
Encourage, support and promote breastfeeding.	Not Scored; subjects not pregnant or breast feeding	
Care for your food; prepare and store it safely.	Not Scored; not adequately measured	

[a] Servings unless otherwise indicated. Participants with intakes between the maximum and minimum amount were assigned scores proportionately.
[b] No quantitative Australian guidelines currently exist. Maximum score cut-offs are based on response categories of short question on water intake in TXT2BFiT baseline survey.
[c] Scored based on total sugar intake as DQESv2 does not measure intake of added sugars, maximum cut-offs are based on the World Health Organization (WHO) guidelines for total sugars informed by two systematic reviews [1,2]

References

1. Te Morenga L, Mallard S, Mann J. Dietary sugars and body weight: systematic review and meta-analyses of randomised controlled trials and cohort studies. BMJ. 2013;346:e7492. DOI: http://dx.doi.org.ezproxy1.library.usyd.edu.au/10.1136/bmj.e7492
2. Moynihan P, Kelly S. Effect on Caries of Restricting Sugars Intake Systematic Review to Inform WHO Guidelines. J Dent Res.2014;93(1): 8–18. DOI: 10.1177/0022034514526408
